# Influence of Welding Speeds on the Morphology, Mechanical Properties, and Microstructure of 2205 DSS Welded Joint by K-TIG Welding

**DOI:** 10.3390/ma14123426

**Published:** 2021-06-21

**Authors:** Shuwan Cui, Shuwen Pang, Dangqing Pang, Zhiqing Zhang

**Affiliations:** 1 School of Mechanical and Transportation Engineering, Guangxi University of Science and Technology, Liuzhou 545006, China; pangshuwen273437@163.com (S.P.); zhangzhiqing@gxust.edu.cn (Z.Z.); 2 Guangxi Zhuang Autonomous Region Tobacco Company Liuzhou Tobacco Company, Liuzhou 545006, China; gxlzpdq@163.com

**Keywords:** K-TIG welding, weld morphology, grain boundary characteristics, microhardness, tensile strength

## Abstract

In this paper, 8.0 mm thickness 2205 duplex stainless steel (DSS) workpieces were welded with a keyhole tungsten inert gas (K-TIG) welding system under different welding speeds. After welding, the morphologies of the welds under different welding speed conditions were compared and analyzed. The microstructure, two-phase ratio of austenite/ferrite, and grain boundary characteristics of the welded joints were studied, and the microhardness and tensile properties of the welded joints were tested. The results show that the welding speed has a significant effect on the weld morphology, the two-phase ratio, grain boundary misorientation angle (GBMA), and mechanical properties of the welded joint. When the welding speed increased from 280 mm/min to 340 mm/min, the austenite content and the two-phase ratio in the weld metal zone (WMZ) decreased. However, the ferrite content in the WMZ increased. The proportion of the Σ3 coincident site lattice grain boundary (CSLGB) decreased as the welding speed increased, which has no significant effect on the tensile strength of welded joints. The microhardness of the WMZ and the tensile strength of the welded joint gradually increased when the welding speed was 280–340 mm/min. The 2205 DSS K-TIG welded joints have good plasticity.

## 1. Introduction

2205 Duplex Stainless Steel (DSS) is a functional integrated material with high strength and excellent corrosion resistance. It is composed of two phases of ferrite and austenite, and the content of both is close to 1:1 [1,2]. 2205 DSS takes into account the advantages of both ferrite and austenite. In recent years, it has been widely used in papermaking, construction, structural materials, nuclear reactors, the petrochemical industry, and underwater engineering. As an important thermal processing technology in manufacturing, welding can change the two-phase structure balance in the 2205 DSS welded joint, which affects the performance of the welded joint [3,4,5,6]. Therefore, the organization and control of the welding process of 2205 DSS has received extensive attention from many disciplines.

At present, in the medium-thick 2205 DSS plate welding process, the multi-layer and multi-pass welding process is often used. It is a traditional welding method, which has low work efficiency and easily destroys the two-phase balance of the heat-affected zone (HAZ). High-energy beam welding is an ideal method for welding medium-thick metal plates. It has high welding efficiency and excellent welding seam shape. The welding heat input required for high-energy beam welding is relatively low, so the cooling rate is faster. The austenite content in the weld metal zone (WMZ) was too low, and coarse ferrite grains were formed, and the grain boundaries are easily corroded. Under low temperature conditions, the WMZ is prone to embrittlement, and the toughness and corrosion resistance of the WMZ decrease. Westin [7] et al. applied CO_2_ laser welding to weld 2205 duplex stainless steel. Coarse ferrite grains formed in the 2205 duplex stainless steel welded joint. Excessive ferrite content and coarse grain structure seriously affect the toughness and corrosion resistance of the welded joint. Zhang [8] et al. pointed out that the two-phase structure was unbalanced in the WMZ of the S31803 DSS electron beam-welded joint, and there is a Cr_2_N precipitated phase. Singh [9] et al. used electron beam welding to weld 18 mm and 12 mm thick 2205 DSS steel plates. They reported that the fine-grained fusion zone with a higher γ/α ratio showed better impact toughness, while the coarser-grained fusion zone with a higher α/γ ratio showed better fatigue properties. The above research shows that the microstructure and mechanical properties of the welded joints are different due to the difference in the cooling rate corresponding to different high-energy beam welding methods. The current research mainly focuses on the effect of cooling rate on the microstructure of the 2205 DSS welded joint and lacks the mechanism analysis of the microstructure evolution during the welding process.

Keyhole tungsten inert gas (K-TIG) welding is a kind of high-energy beam welding method. It can be welded in a single-pass welding and formed on both sides without filling the welding material [10,11,12,13,14]. In this paper, different welding speeds were applied to analyze the influence of cooling rate on morphology, mechanical properties, and microstructure of the 2205 DSS welds. The evolution law of the two-phase ratio, grain boundary misorientation angle (GBMA), and mechanical properties of the welded joints under different welding speeds were studied.

## 2. Materials and Methods

### 2.1. Material and Welding Procedure

**Figure 1:**
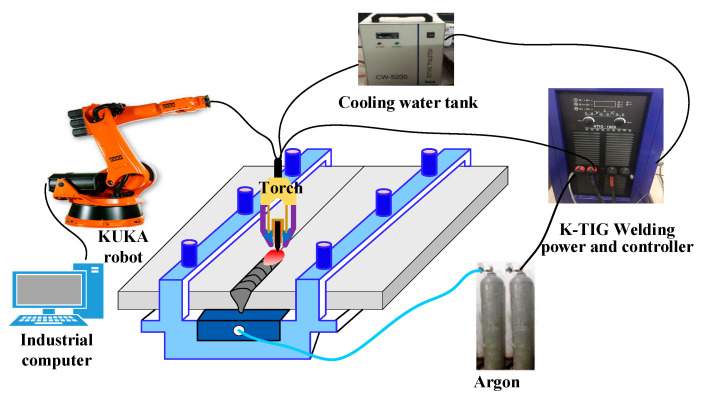
Schematic diagram of keyhole tungsten inert gas (K-TIG) welding.

Figure 1 shows the schematic diagram of K-TIG welding. The butt-joint welding method was adopted, the workpieces to be welded were fixed before welding, and there was no obvious wrong edge and gap between the workpieces. The high-purity argon was applied as the protective gas on the front and back of the workpieces, and the gas flow rate was 25 L/min. The specific welding parameters are shown in Table 1. The 2205 DSS plates with a thickness of 8 mm were selected as the base material (BM), and its chemical composition was shown in Table 2. The dimensions of the BM were 300 mm × 100 mm.

### 2.2. Mechanical Properties Test and Microstructure

**Figure 2:**
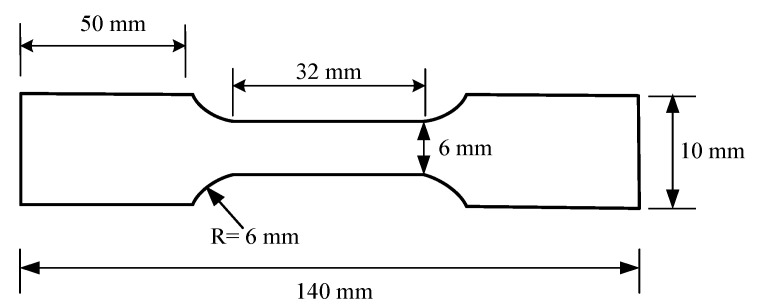
Tensile test specimen dimension.

The Vickers microhardness tests were performed on the cross section of the 2205 DSS welded joints. During the test, a load of 100 g and a dwell time of 10 s were used to measure the microhardness. The tensile test specimens of the 2205 DSS welded joints were provided according to the American society of testing materials (ASTM) E8 [15], the tensile test specimen dimension was shown in Figure 2. For each set of parameters, two tensile specimens are taken for testing, and the strength and elongation were averaged. A scanning electron microscope (SEM) was applied to observe the morphologies of the tensile fracture after tensile tests.

The microstructure of 2205 DSS welded joints were observed by using optical microscope (OM). 180#, 240#, 400#, 600#, 800#, 1000#, 1200#, 1500#, and 2000# water sandpapers were used to grind the cross section of the welded joint step by step. Finally, a 2 μm diamond polishing agent was used for mechanical polishing on the polishing machine. Beraha etchant (30 mL H_2_O + 60 mL HCL + 1 g K_2_S_2_O_5_) was applied to etch the polished sample, and the etch time was ~11 s [16]. We rinsed the welded joints with water after corrosion and then dried them with a hair dryer for observation of their microstructure. According to ASTM E1245-03 (2016) standard, the austenite content, ferrite content, and two-phase ratio were analyzed using Image Pro software [17]. The characteristics of the grain boundary was characterized by electron backscatter diffraction (EBSD) technique. 

## 3. Results and Discussion

### 3.1. Influence of Welding Speed on the Weld Morphology

**Figure 3:**
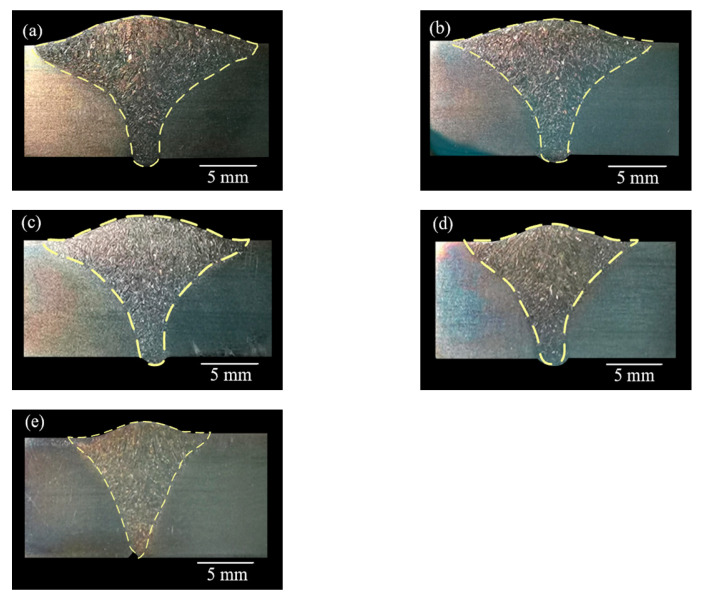
Cross section of 2205 DSS K-TIG welded joints at different weld speeds: (**a**) 280 mm/min, (**b**) 300 mm/min, (**c**) 320 mm/min, (**d**) 340 mm/min, and (**e**) 360 mm/min.

**Figure 4:**
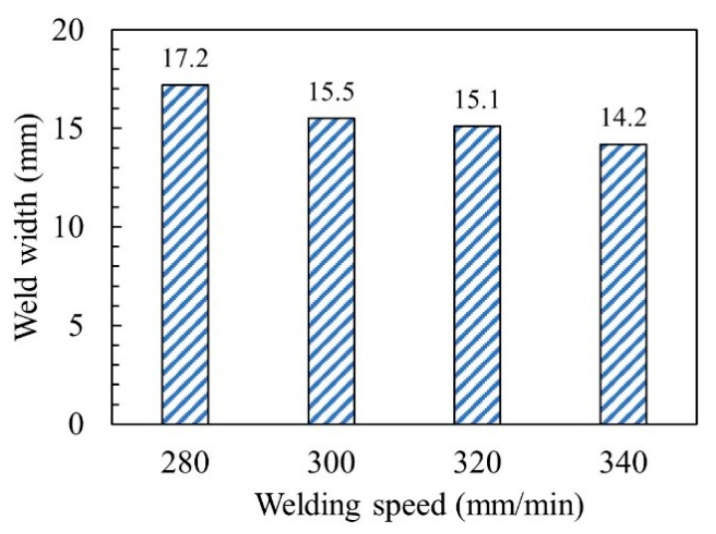
Weld width at different welding speeds.

There were obvious differences in the morphology of the weld under different welding speed conditions, as shown in Figure 3. When the welding speed was 280–340 mm/min, the front and back sides of the welds have reinforcements, the weld seams were well formed, and the cross section of the welds were nail-shaped. As the welding speed increased, the head of the nail-shaped gradually narrow and short, that was, the weld width gradually decreased, as shown in Figure 4. When the welding speed was 360 mm/min, the cross section of the weld was triangular, and the workpieces were not completely penetrated. This was because the higher welding speed results in a lack of welding heat input, and the arc penetration ability was weakened. It can be seen that the welding speed can significantly affect the morphology of the weld.

### 3.2. Microstructure and Two-Phase Ratio in the WMZ

**Figure 5:**
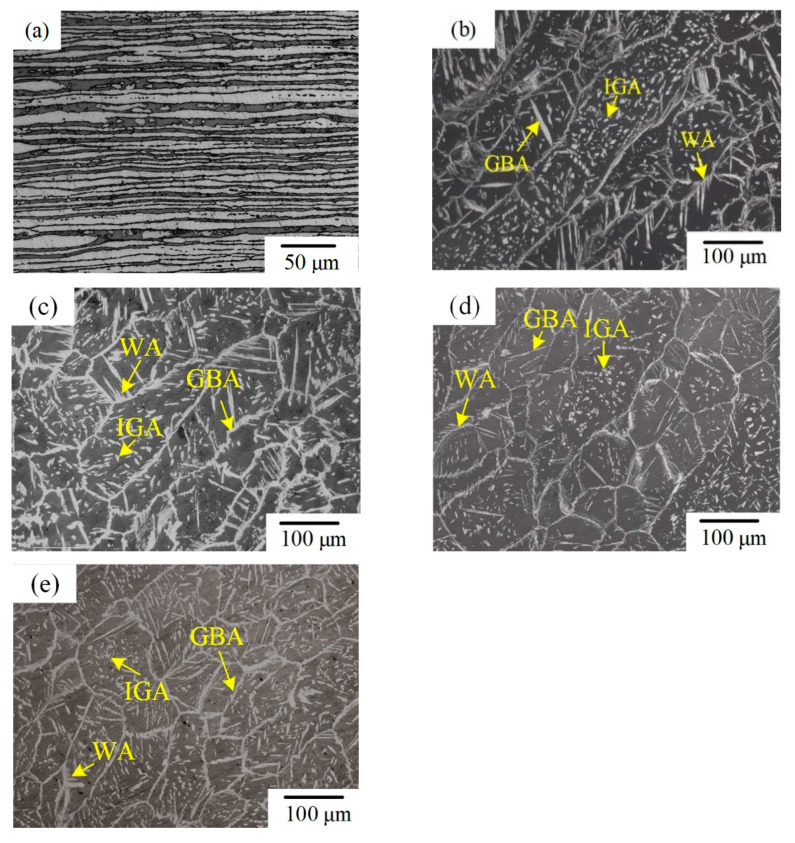
Microstructure of 2205 DSS K-TIG welded joint. (**a**) BM. WMZ under different welding speeds (mm/min): (**b**) 280, (**c**) 300, (**d**) 320, and (**e**) 340.

**Figure 6:**
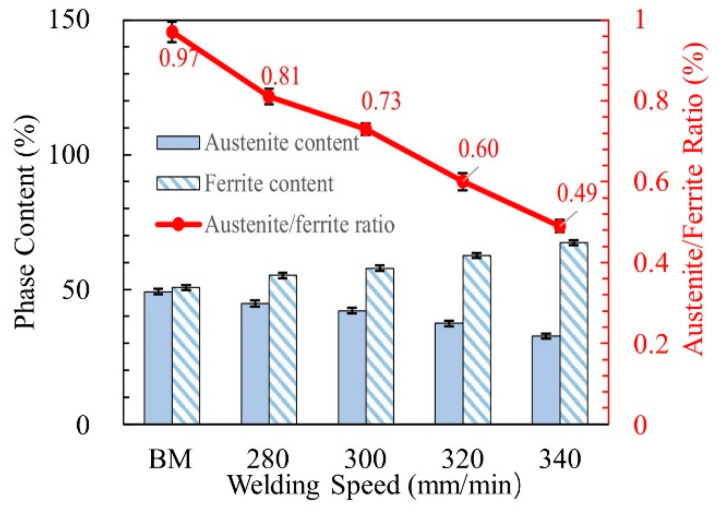
Phase content and austenite/ferrite ratio in the BM and WMZ under different welding speeds.

When the welding speed was 360 mm/min, the workpieces were not completely penetrated, which did not meet the welding requirements. Therefore, this was conducted only to study the microstructure and property of the welded joint when the welding speed was from 280 to 340 mm/min. Figure 5 shows the microstructure of the BM and WMZ at different welding speeds. The BM was composed of austenite and ferrite. The light phase was austenite and the dark phase was ferrite; the austenite was distributed on the ferrite matrix in the form of long strips, as shown in Figure 5a. When the welding speeds were 280–340 mm/min, the austenite in the WMZ exists in three forms: intergranular austenite (IGA), grain boundary austenite (GBA), and Widmanstätten austenite (WA). The ferrite in the WMZ mainly came from the solidification process of the liquid phase, while the austenite was mainly produced from the solid phase transformation process of ferrite. The arrangement of atoms at the ferrite grain boundary was disordered, and the free energy was relatively high. Austenite precipitates at the grain boundary to form GBA. As the cooling progresses, the austenite content at the grain boundary increased, and the location at the boundary that was beneficial for nucleation decreased. Therefore, austenite grew rapidly into the ferrite grains in parallel laths at the boundary, so it formed WA. As the temperature decreases, IGA gradually forms. Figure 6 displayed the austenite content, ferrite content and the two-phase ratios of the BM and the WMZ under different welding speeds. When the welding speed was 280–340 mm/min, the ferrite content of the WMZ under different welding speeds were significantly higher than that of the BM. The ferrite is a body-centered cubic structure and austenite is a face-centered cubic structure. Therefore, the welds without filling metal have reinforcements on the front and back sides. With the increased of welding speed, the content of austenite and the two-phase ratio in the WMZ gradually decreased, and the content of ferrite gradually increased. This was mainly because as the welding speed increased, the welding cooling rate increased, and austenite did not have enough time to precipitated from the ferrite.

### 3.3. Grain Boundary Characteristics 

Park et al. proved that the grain boundaries have a very important influence on the mechanical and physical properties of polycrystalline materials [18]. Previous studies showed that the difference in the GBMA can cause the material to produce completely different deformation behaviors when the material was deformed [19]. In the process of deformation, high-angle grain boundaries (HAGB; misorientation angle greater than 10°) can completely hinder the movement of dislocations [20,21,22]. However, the low-angle grain boundaries (LAGB; misorientation angle less than 5°) can react with moving dislocations to form new dislocations. Compared with the LAGB, HAGB mainly made crack propagation more difficult. Therefore, whether the material has good toughness depends on the proportion of HAGB. Under the same conditions of other parameters, the higher the proportion of HAGB, the better the toughness of the material.

**Figure 7:**
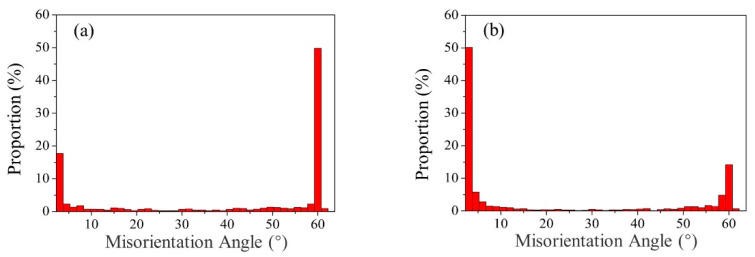

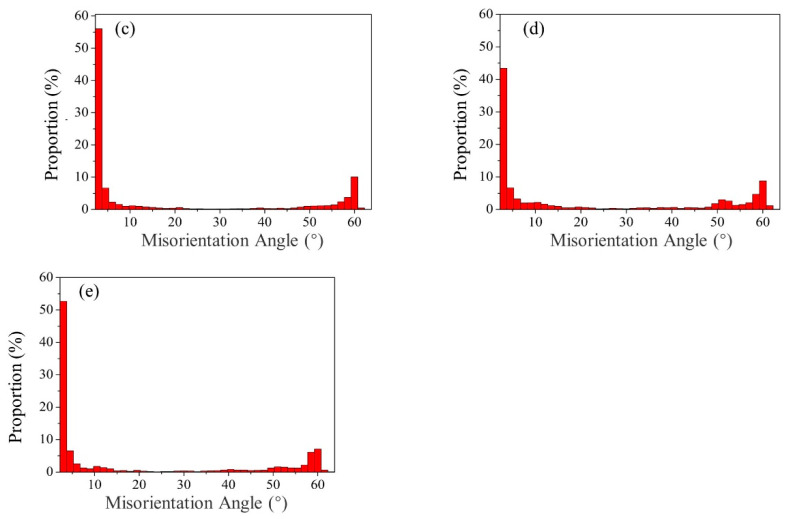
GBMA distribution maps of the welded joint. (**a**) BM. WMZ under different welding speeds (mm/min): (**b**) 280, (**c**) 300, (**d**) 320, and (**e**) 340.

Figure 7 shows the GBMA distribution maps of the BM and WM under different welding speeds. In the BM, the GBMA was mainly distributed in HAGB. The proportion of the Σ3 coincident site lattice grain boundary (CSLGB, misorientation angle equal to 60°) was 49.8%, as shown in Figure 7a. Figure 7b–e displays the GBMA distribution maps of the WM. It can be seen that in all the WMZ, the GBMA were mainly distributed in LAGB. The proportion of the Σ3 CSLGB in the WMZ under different welding speeds were 14.2%, 10.1%, 8.8%, and 7.1%, respectively. It proved that the HAGB in the WMZ have migrated to LAGB after K-TIG welding, and part of the Σ3 CSLGB has been consumed during the migration process.

### 3.4. Mechanical Properties

#### 3.4.1. Microhardness 

**Figure 8:**
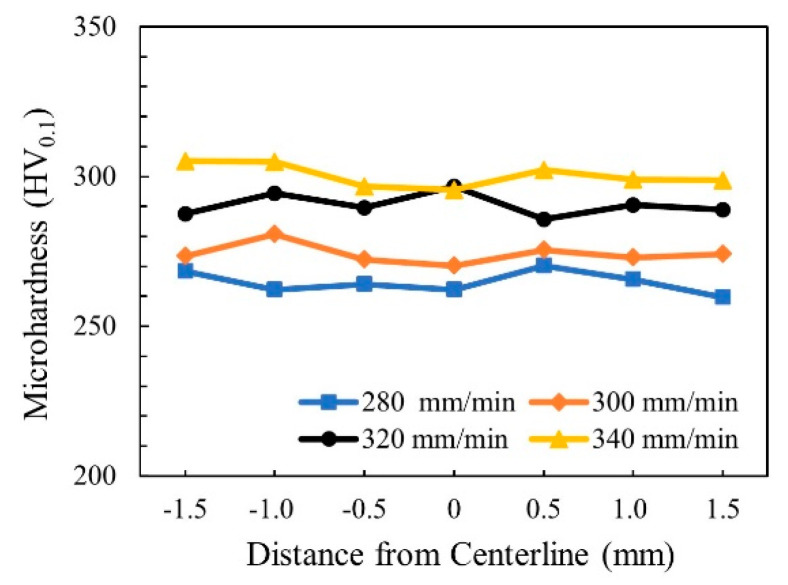
Microhardness distribution trend map of WMZ under different welding speeds.

Figure 8 shows the microhardness distribution trend map of WMZ under different welding speeds. Take the center line of the weld as the axis of symmetry, and take three points on each side, with an interval of 0.5 mm between each point. When the welding speeds were 280–340 mm/min, the average value of the microhardness of the WMZ were 264.6 ± 3.7 HV, 274.1 ± 3.4 HV, 290.5 ± 3.8 HV, and 300.3 ± 3.8 HV, respectively. However, the average value of the microhardness of the BM was 229.4 ± 2.6 HV. The microhardness of all WMZ were higher than that of the BM. This was because the two-phase ratio of austenite/ferrite in the WMZ decreased significantly after welding. In the microstructure of 2205 DSS, the microhardness of ferrite is greater than that of austenite. Therefore, the microhardness of the 2205 DSS K-TIG WMZ was inversely proportional to the two-phase ratio of austenite/ferrite. The lower the two-phase ratio of austenite/ferrite, the higher the microhardness corresponding to the area. Figure 6 shows that as the welding speed increases, the two-phase ratio of austenite/ferrite gradually decreases. Therefore, the microhardness of the WMZ increased with the increase of welding speed.

#### 3.4.2. Tensile Property

Table 3 displays the tensile test results of 2205 DSS BM and K-TIG welded joints under different welding speeds. The tensile strength of the BM was 804.0 ± 2.12 MPa. Under different welding speed conditions, the tensile strength of 2205 DSS K-TIG welded joints were significantly different. When the welding speeds were 280–320 mm/min, the tensile strength of the welded joints gradually decreased, and the tensile fracture positions were all in the WMZ. It can be seen that when the welding speeds were 280–320 mm/min, the tensile strength of WMZ in the welded joints were lower than that of the BM. This was mainly because the grains of the WMZ grew up under the action of the welding thermal cycle. The coarse grain size can also reduce the tensile strength of the welded joint. When the welding speed was increased to 340 mm/min, the tensile strength of the welded joint was 800.3 ± 1.76 MPa, and the tensile fracture position was the BM, which was far away from WMZ. At this time, the tensile strength of the WMZ in the welded joint was significantly greater than that of the BM. Therefore, the tensile fracture position occurs in the BM. This was because as the welding speed increased, the content of ferrite in the WMZ increased, and the content of austenite decreased. As ferrite has a body-centered cubic structure, the ferrite in the DSS determined the tensile strength of the material. In addition, as the welding speed increased, the cooling rate increased, and the grains in the WMZ did not have enough time to grow. Therefore, the tensile strength of the WMZ gradually increased as the welding speed increased. Combining the characteristics of the grain boundary in the WMZ under different welding speeds, it can be seen that the increased of the LAGB proportion and decreased of the Σ3 CSLGB proportion no significant effect on the tensile strength of the welded joint.

**Figure 9:**
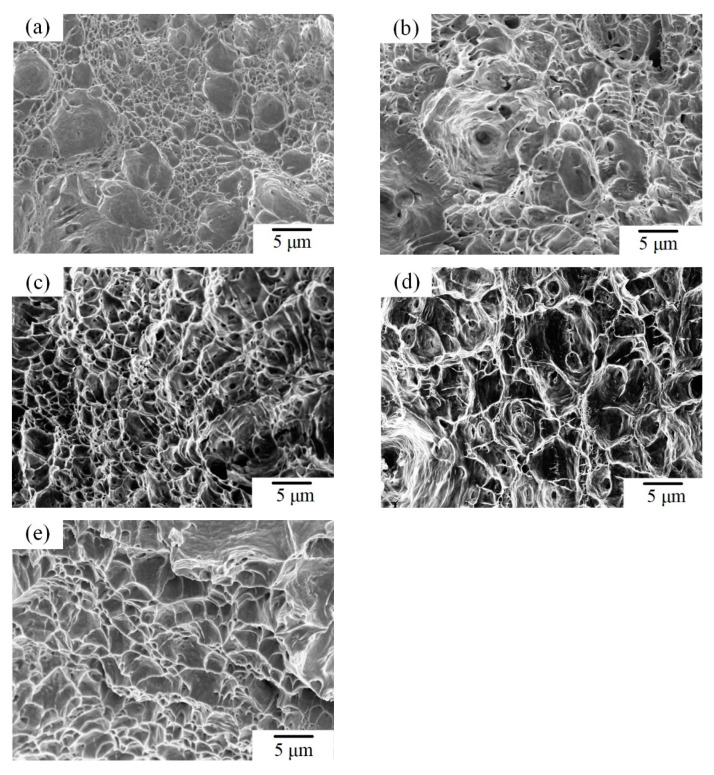
SEM image of tensile fracture. (**a**) BM. Welded joints under different welding speeds (mm/min): (**b**) 280, (**c**) 300, (**d**) 320, and (**e**) 340.

The elongation of the BM was 46.6 ± 0.71%. when the welding speeds were 280–340 mm/min, the elongation of the welded joint were 27.9 ± 0.99%, 24.7 ± 0.99%, 23.1 ± 0.42%, and 32.3 ± 0.57%, respectively. When the welding speeds were 280–320 mm/min, the elongation of welded joints were gradually reduced. This showed that when the welding speeds were 280–320 mm/min, the plasticity of the welded joint decreased as the welding speed increased. However, when the welding speed was 340 mm/min, the elongation of the welded joint was larger. It was mainly because when the welding speed was 340 mm/min, the tensile strength of WMZ was better than that of the BM, so the tensile fracture position occurred in the BM. Therefore, the amount of deformation of the welded joint during the tensile process was different. At this time, the elongation cannot be used to compare and analyze the plasticity of welded joint. Figure 9a shows the morphologies of tensile fracture of BM. The tensile fracture surface of the BM has a typical ductile fracture morphology. Figure 9b,c shows the morphologies of tensile fracture of WMZ in the welded joints under welding speeds were 280–320 mm/min. Figure 9e shows the morphology of tensile fracture of BM in the weld joint when the welding speed was 340 mm/min. When the welding speeds were different, the tensile fracture positions of the welded joints were different. However, all the tensile fracture surfaces of the welded joints showed dimples and tearing edges, which indicated that plastic deformation occurred at the fracture positions of the welded joints before the tensile fracture. Therefore, it can be explained that the 2205 DSS K-TIG welded joints have good plasticity.

## 4. Conclusions

1. When the welding speeds were 280–340 mm/min, the front and back sides of the 2205 DSS welds were well formed.
2. Under different welding speeds, the austenite morphologies of the WMZ mainly exist in three forms: WA, GBA, and IGA. The content of austenite and the ratio of the two-phase in the WMZ decreased, the content of ferrite in the WMZ increased as the welding speeds increased.
3. When the welding speeds were 280–340 mm/min, the proportion of Σ3 CSLGB decreased as the welding speed increased. However, the change of Σ3 CSLGB proportion has no significant effect on the tensile strength of welded joints.
4. When the welding speed increased from 280 mm/min to 340 mm/min, the microhardness of the WMZ and the tensile strength of the welded joint gradually increased. The 2205 DSS K-TIG welded joints have good plasticity.

## Figures and Tables

**Table 1:** Test parameters.

Welding Parameters	Value
Weld current	480 (A)
Arc voltage	16.7 (V)
Weld speed	280, 300, 320, 340, 360 (mm/min)
Diameter of tungsten electrode	6.4 (mm)
Electrode gap	2.5 (mm)
Shielding gas	Pure argon (99.9%)

**Table 2:** Chemical composition of the base material (BM).

Element	Cr	Ni	Mn	Mo	Si	C	N	S	P	Fe
Wt. %	22.46	5.70	1.428	3.02	0.057	0.019	0.156	0.0005	0.021	Bal.

**Table 3:** Tensile test results of the 2205 DSS BM and welded joint.

Test Area	Welding Speed (mm/min)	Tensile Strength (MPa)	Elongation (%)	Tensile Fracture Position
Weld joint	280	752.3 ± 1.98	27.9 ± 0.99	WMZ
	300	789.5 ± 1.34	24.7 ± 0.99	WMZ
	320	794.5 ± 1.69	23.1 ± 0.42	WMZ
	340	800.3 ± 1.76	32.3 ± 0.57	BM
BM	/	804.0 ± 2.12	46.6 ± 0.71	/

## Data Availability

Data sharing is not applicable to this article.
